# Decades of cholera in Odisha, India (1993–2015): lessons learned and the ways forward

**DOI:** 10.1017/S0950268821001266

**Published:** 2021-06-07

**Authors:** Hemant Kumar Khuntia, Thandavarayan Ramamurthy, Madhusmita Bal, Sanghamitra Pati, Manoranjan Ranjit

**Affiliations:** 1 ICMR-Regional Medical Research Centre, Chandrasekharpur, Bhubaneswar, Odisha, India; 2 ICMR-National Institute of Cholera and Enteric Diseases, P-33, CIT Road, Scheme XM, Beleghata, Kolkata, 700010, India

**Keywords:** Antibiotics, cholera, Odisha, variant, *Vibrio cholerae*

## Abstract

Cholera is one of the major public health problems in the state of Odisha, India since centuries. The current paper is a comprehensive report on epidemiology of cholera in Odisha, which was documented from 1993. PubMed and Web of Knowledge were searched for publications reporting cholera in Odisha during the period 1993–2015. The search was performed using the keywords ‘Odisha’ and/or ‘Orissa’ and ‘Cholera’. In addition, manual search was undertaken to find out relevant papers. During the study period, a total of 37 cholera outbreaks were reported with an average of >1.5 cholera outbreaks per year and case fatality ratio was 0.3%. *Vibrio cholerae* O1 Ogawa serotype was the major causative agent in most of the cholera cases. The recent studies demonstrated the prevalence of *V. cholerae* O1, El Tor variants carrying *ctxB1*, *ctxB7* and Haitian variant *tcpA* allele associated with polymyxin B sensitivity and these variants are replacing the proto type El Tor. The first report of variant *ctxB7* in Odisha during super-cyclone 1999 predicted its emergence and subsequent spread causing cholera outbreaks. The prevalence of multidrug-resistant *V. cholera*e at different time periods created alarming situation. The efficacy trial of oral cholera vaccine (OCV, Shanchol) in a public health set-up in Odisha has shown encouraging results which should be deployed for community level vaccination among the vulnerable population. This paper has taken an effort to disseminate the valuable information of epidemiology of cholera that will influence the policy-makers and epidemiologists for constant surveillance in other parts of Odisha, India and around the globe.

## Introduction

Despite considerable advances in the field of epidemiology, diagnosis and management, cholera still remains as an important cause of morbidity and mortality in the developing countries. It affects mostly the people, with limited access to safe drinking-water supply and inadequate sanitation. The World Health Organization (WHO) estimates that globally there are about 3–5 million cholera cases and 100 000–120 000 deaths every year, out of which only a small fraction is officially reported [1]. The number of cholera cases reported to WHO has continued to be high over the last few years. During 2019, 923 037 cases, 1911 deaths were notified from 31 countries [2]. An estimated 2.86 million cholera cases (uncertainty range: 1.3 –4.0 m) occur annually in endemic countries. Among these cases there are an estimated 95 000 deaths (uncertainty range: 21 000–143 000) [3].

Cholera is characterised by sudden onset and painless passage of a large volume of rice watery stool, leading to severe dehydration, and becomes life threatening in the absence of prompt treatment [4, 5]. *Vibrio cholerae*, a Gram-negative bacterium is the causative agent of cholera [6]. Based on somatic ‘O’ antigen, more than 206 serogroups of *V. cholerae* has been identified and among them, *V. cholerae* O1 and O139 are the epidemic serogroups [7]. *V. cholerae* O1 has two major serotypes, Ogawa and Inaba. These serotypes can further be distinguished into two biotypes i.e. classical and El Tor [8] based on certain biochemical properties and susceptibility to *V. cholerae* O1-specific bacteriophages. Currently, *V. cholerae* O1 is the most prevailing serogroup in cholera-endemic countries, after a considerable decline of the serogroup O139 over the past few years [9]. Since late ninety, *V. cholerae* O1 El Tor biotype is appearing in new forms with cryptic genetic changes [10–15]. According to the recently redefined biotype scheme, *V. cholerae* O1 strains carrying mixed phenotypes of classical and El Tor biotypes are designated as hybrid biotypes, whereas *V. cholerae* O1 strains with typical El Tor phenotypes, but carrying classical *ctxB* (the gene that encodes cholera toxin B1 subunit) are considered as El Tor variant [16]. The classical *ctxB* gene in El Tor variant has undergone mutation, which has been named as *ctxB* genotype 7 [14]. In 2010 a large outbreak was occurred in Haiti caused by this new *V. cholerae* El Tor variant harbouring *ctxB*7. Subsequently this El Tor variant was highlighted as Haitian variant. Recent investigations revealed that this new El Tor variant with *ctxB*7 originated in West Bengal during 2006 [17] and progressively disseminated to other states/countries [18–21]. Globally, Haitian variant is spreading fast, replacing the prototype El Tor biotype, hybrid and El Tor variant *V. cholerae* O1 carrying *ctxB1*.

Odisha (formerly known as Orissa) is located along the Bay of Bengal between 17.78°N to 22.73°N latitudes and 81.37°E to 87.53°E longitudes. The state has an area of 155 707 km^2^ and comprised of 30 administrative districts. The total population of the state is about 42 million and 83.3% of them live in rural areas. As per the record of the Government of Odisha (October 2016), out of the total 156 468 rural habitations in the state around 25% have a safe drinking-water supply and 34% have proper toilet facilities. Majority of the population in rural areas, use surface water for drinking, bathing, cooking and irrigation. As evident from news excerpters and official gazettes, the state experiences outbreaks of cholera almost every year after natural disasters like floods and cyclones and mass gathering for different festivals like car festivals of Puri.

Bay of Bengal is a hot spot for cyclones and coastal Odisha is vulnerable for tropical cyclone landfall. Between 1891 and 2019, the state was hit by about 111 cyclones. Odisha has witnessed seven major cyclones in the last 50 years among which Super-cyclone, 1999 and Failin in 2019 are strongest. The unprecedented super-cyclone with high wind velocity caused sea water invasion up to 30 km inland followed by a large cholera outbreak [22]. It was presumed that there might be the emergence of a new serogroup or at least new clones of *V. cholerae* involved in the outbreak as the marine milieu highly favours the vibrios for survival and proliferation [23].

Recently, the high protective efficacy of an oral cholera vaccine (OCV, Shanchol) has been tested in a public health set-up in Odisha [24]. This has facilitated the health officials to consider OCV as one of the management strategies for preventing cholera in future. Systematic scientific study on the current situation of cholera in the state has not been performed. In this report, using several published scientific papers and other records, we have documented several cholera outbreaks, their distribution, phenotypic and genetic changes of *V. cholerae*, drug resistance, vaccination outcomes, etc., mainly to help the health authorities and policy-makers to adopt useful control strategies in prevention and spread of cholera.

## Methods

We searched PubMed and Web of Knowledge from 1 January 1993 to 31 December 2015 following the scoping review approach. We did not search before the year 1993, because our goal was to help in the decision-making rather than providing a historical perspective of the disease. We searched the existing literature using the key words ‘Odisha’ and/or Orissa with ‘*V. cholerae*’ and ‘Cholera’ and identified 35 relevant publications. To identify additional studies, reference lists of publications were carefully screened. We have also included data available in the annual reports of the Institutes/Departments. Most of the data included here are from publications of the ICMR-Regional Medical Research Center, Bhubaneswar; Department of Health Services, Govt. of Odisha; Srirama Chandra Bhanja Medical College and Hospitals, Cuttack; Maharaja Krishna Chandra Gajapati Medical College & Hospitals, Brahmapur, Ganjam; ICMR-National Institute of Cholera and Enteric Diseases, Kolkata; Defense Research & Development Establishment (DRDE), Gwalior and DBT – Institute of Life Science, Bhubaneswar.

To identify cholera cases in districts of Odisha where cholera cases occurred, we included major reports of routine surveillance activities and outbreaks published in articles and annual reports of RMRC, Bhubaneswar. A routine surveillance system is defined as any mechanism exists in hospital or institute for purpose to identify pathogens responsible for causing diarrhoea using microbiological techniques. Thus all cholera cases identified during routine surveillance were confirmed by bacterial culture. During outbreaks representative stool samples from diarrhoea patients were cultured and confirmed by standard laboratory tests for *V. cholerae* O1 and *V. cholerae* O139. An outbreak was defined as the occurrence of more cholera cases than expected during a specific period.

According to WHO, a cholera-endemic area is an area where confirmed cholera cases were detected during the last 3 out of 5 years with evidence of local transmission. In an endemic area the causative organism reside in a local aquatic environment and the occurrence of disease in human is quite independent without being imported from outside. The endemic of cholera depends on the environmental reservoir of *V. cholerae*. Cholera outbreaks occur due to interplay of favourable climatic conditions and poor sanitation.

RMRC Bhubaneswar is a leading research institute in the state involves in various biomedical research including diarrhoeal surveillance study and outbreak investigation. Besides these, RMRC is called upon by the Ministry of Health and Family Welfare Department, Government of Odisha to investigate cholera outbreaks occurring in the state. The data of surveillance and outbreak investigations are published in annual reports of RMRC and published in national and international journals.

From 35 identified published articles, RMRC annual reports and other sources we extracted data related to cholera, including year of outbreak, area affected, number of cholera cases diagnosed, number of deaths due to cholera, types of *V. cholerae* strain isolated, antibiotic profile of *V. cholerae* strains, methods for control, cause of outbreaks and sanitation and water supply system in the affected areas.

## Results

**Fig. 1.**
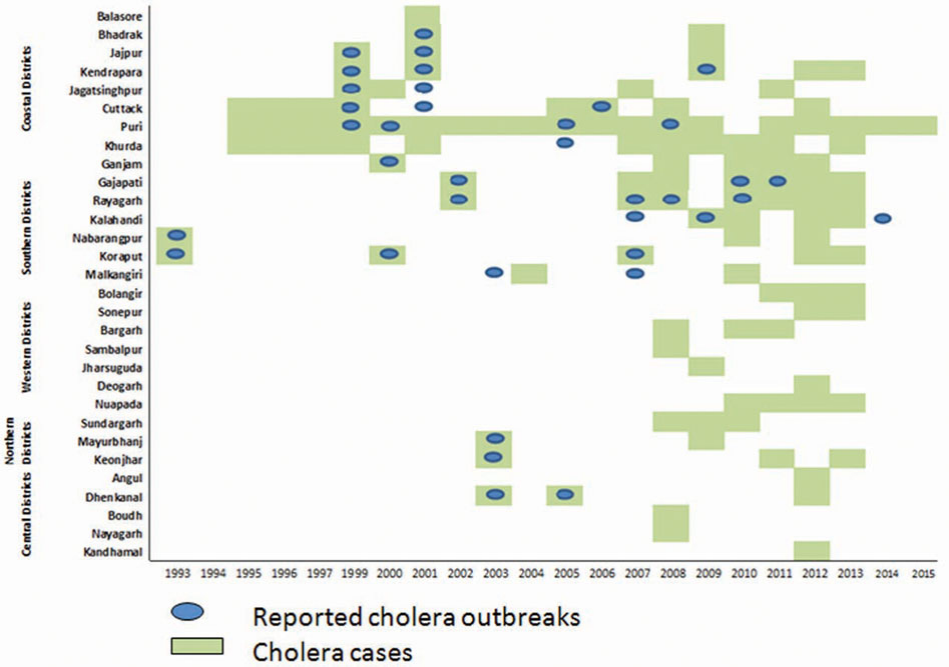
Distribution of cholera cases and outbreaks in Odisha, 1993–2015.

A total number of 35 research articles were published during 1993–2015, besides the annual reports of different institutes of the state on outbreak investigations and/or routine clinical investigations, surveillance reports and antibiogram patterns. From the available literatures/reports, it is evident that all the 30 districts have reported either sporadic cases or epidemic outbreaks of cholera at singular and/or regular point of time (Fig. 1). A total of 37 outbreaks of cholera (average more than 1.5 per year) have been reported during 1993–2015 affecting around 90 773 individuals (≈4323 per year) and causing 275 deaths (≈13 per year) (Table 1). The number of affected individuals reported here ranged from as low as 41 [25] and high of 123 546 [26]. The maximum number of cholera cases was reported during October 1999, after the super-cyclone that has caused 55 deaths, mostly in coastal areas [22]. The case fatality ratio (range 13.04–0.1) was found to be little higher than the other reports [9]. Interestingly, a decreasing trend in the mortality rate has been detected since 2002. In the 21-year study period with 275 deaths due to cholera, the overall case fatality ratio was 0.3%.

**Table 1.** Outbreaks, cholera cases and deaths from 1993 to 2014 in Odisha

Year	No. of outbreaks	Cholera cases	Deaths	CFR%
1993	2	34	–	–
1999	5	32 039	55	12.41
2000	3	443	–	–
2001	5	147	–	–
2002	2	23	03	13.04
2003	4	41	–	–
2005	3	175	01	0.57
2006	1	146	–	–
2007	4	8206	162	1.97
2008	2	44	–	–
2009	2	715	07	0.98
2010	2	2288	43	1.87
2011	1	236	01	0.4
2014	1	46 236	03	0.006
Total	37	90 773	275	Average CFR: 0.3

**Fig. 2.**
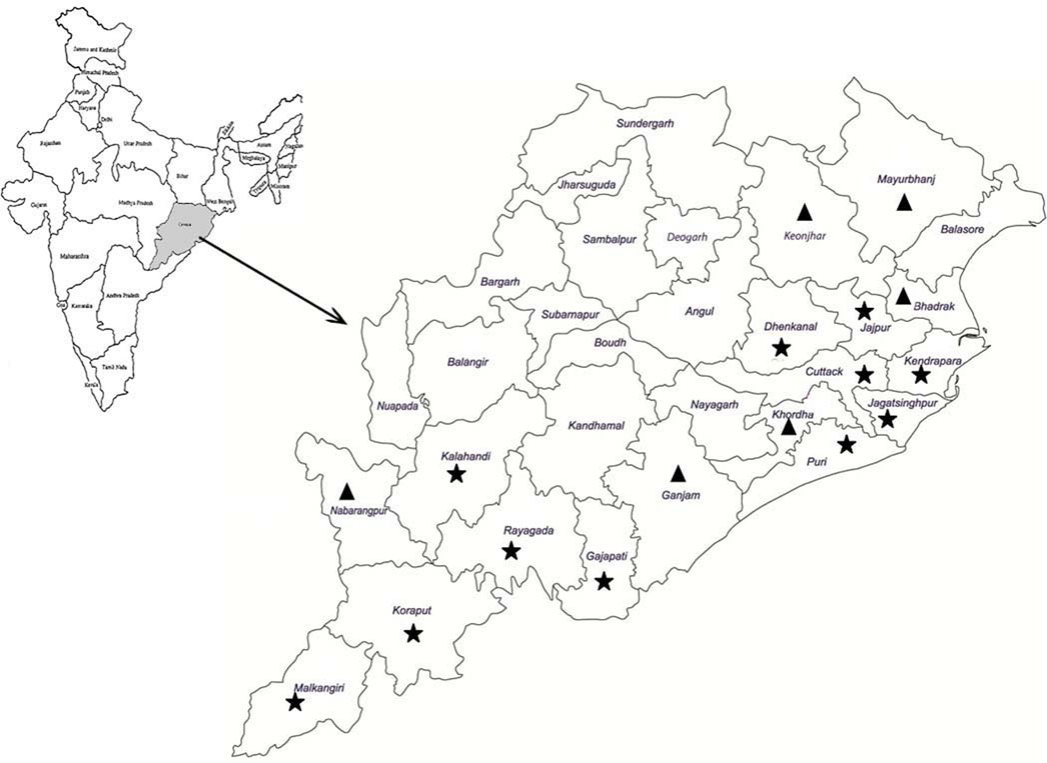
Cholera outbreak districts. ★ Indicates cholera outbreaks ≥2; ▴ indicates one cholera outbreak.

The frequency of epidemic outbreaks of cholera was more in the costal (17 out of 37 outbreaks) districts situated above 0–150 mean sea level (msl) and formed by the deltaic deposits of rivers falling into the Bay of Bengal and tribal dominated southern districts (16 out of 37 outbreaks) situated above 900–1350 msl and formed of steep-sided mountains with canyon and inter mountainous fertile valleys/plateaus compared to districts of northern and western regions situated above 150–900 msl. Of the total outbreaks, coastal and southern districts together contributed 89% of all reported outbreaks. Amongst the coastal districts (total 10 in number), the number of outbreaks were more in Puri (4 out of 17) and Cuttack (3 out of 17) districts, while amongst the tribal dominated districts Rayagada has reported more number of outbreaks (4 out of 16) followed by Kalahandi and Gajapati (3 each out of 16). The sporadic cases of cholera have been reported frequently from Puri followed by Khordha and Cuttack districts in the coastal belt and occasionally from other districts. The districts are stratified into two categories based on the number of outbreaks (Fig. 2). Coastal and southern districts reported a higher number of outbreaks and envisaged at least two cholera outbreaks.

**Fig. 3.**
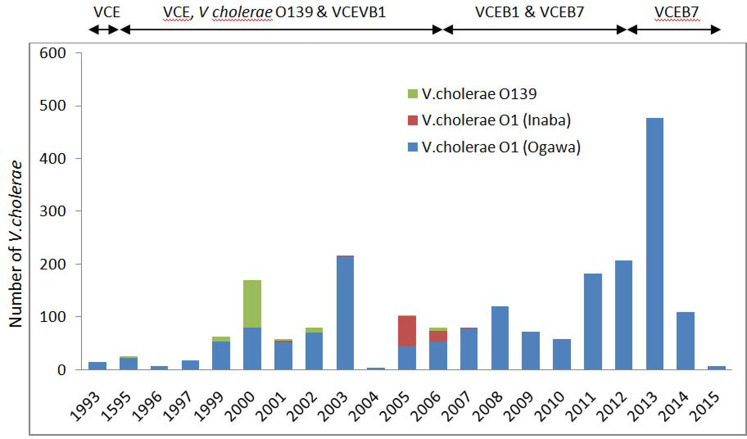
Yearly reported number of cholera cases, by *V. cholerae* O1 and O139 during 1993–2015 in Odisha. VCE, *V. cholerae* O1, El tor biotype VCE; B1, *V. cholerae* O1, El tor biotype with *ctxB1*; VCEB7, *V. cholerae* O1, El tor biotype with *ctxB7*.

Year-wise distribution of the number of reported cholera cases from routine surveillance, referral cases and cholera outbreaks from 1993 to 2015 is shown in Figure 3. Highest number of cholera cases was confirmed during 2013 followed by 2003 and 2012. As a whole a total of 2237 (17.6%) *V. cholerae* has been isolated during the study period from 12 710 diarrhoea cases.

Figure 3 shows the year-wise distribution of biotype and serotype of *V. cholerae* strains for the past 21 years (1993–2015). As reported, *V. cholerae* O1 serotype Ogawa, biotype El Tor was dominant during this period. Amongst all cholera cases, 90.4% were due to serotype Ogawa and 3.9% due to Inaba. The emergence and circulation of *V. cholerae* O1 Inaba was reported from July 2005 to June 2006 [27] followed by a re-emergence of Ogawa strains [28]. *V. cholerae* O139 serogroup was reported to cause cholera for the first time in Odisha during 1995 [29]. This new serogroup remained non-active until 1998, but re-emerged in 1999 [22] and continued till 2006 (≈5.4%) co-existed with *V. cholerae* O1. *V. cholerae* O1, classical biotype was not reported during the study period in Odisha.

Most noteworthy observation was the detection of El Tor variant with *ctxB1* genotype during 2000–2012 [26, 30] and Haitian variants with *ctxB7* detected retrospectively in the super-cyclone strains, 1999 [31]. El Tor variant caused epidemics in tribal dominated southern districts (Raygada, Koraput and Kalahandi) in 2007 [26] that subsequently spread throughout the state till 2012 [30]. Concurrent prevalence of El Tor variant and hybrid strains was also detected during 2008–2009 [30]. Based on the genetic and phenotypic characteristics, three groups of hybrid *V. cholerae* O1 has been reported. Group-1: hybrids were *V. cholerae* O1 with El Tor phenotypes carrying both *ctxB* El Tor and classical gene (*ctxB*^C+E^), group-2: hybrids were polymixin B-sensitive El Tor strains with *ctxB*^C^ and group-3: hybrid strains were polymixin B sensitive El Tor strains with *ctxB*^C+E^. During 2010, El Tor variant with *ctxB1* circulated in this part of the country with the complete replacement of the prototype El Tor [30, 31] and remained dominant till 2014. However, Haitian variant was simultaneously reported with El Tor variant in the same tribal-dominated southern districts during the 2007 outbreak [13] that might have spread to different parts of the state replacing El Tor variant. Haitian variant was observed to cause an outbreak in a tribal district of Kalahandi, in 2014 [32] while in coastal district of Puri, all the *V. cholerae* O1 were reported to harbour Haitian variant *ctxB7* associated with Haitian variant *tcpA* during 2014 and 2015 [33]. Although *V. cholerae* has been found to affect all age groups, but it is more frequent in adults. Outbreaks were found to be associated with low case fatality ratio (0.1%) in coastal districts, but high (13.04%) in tribal districts. The variants of *V. cholerae* affected naïve population of all age groups and spread rapidly.

The seasonality of cholera in Odisha occurs in rainy season which attains its peak during the month of September in each year [34]. The illness of cholera is very often encountered among the young adults and adults while the proportion of cholera is nearly same in males and females (2.1:1.8). The main vehicle of transmission is contaminated water and food [26].

**Fig. 4.**
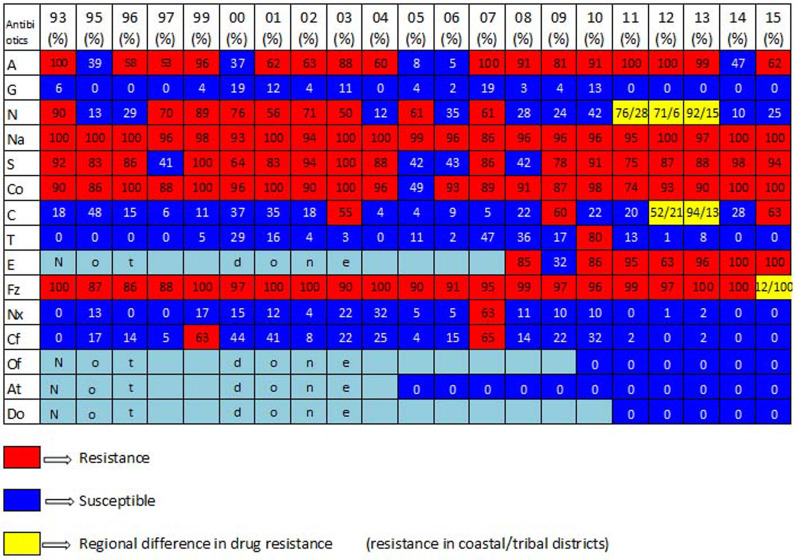
Antibiogram profile of *V. cholerae* O1 during 1993–2015 in Odisha. A, ampicillin; G, gentamycin; N, neomycin; Na, nalidixic acid; S, streptomycin; Co, co-trimoxazole; C, chloramphenicol; T, tetracycline; E, erythromycin; Fz, furazolidone; Nx, norfloxacin; Cf, ciprofloxacin; Of, ofloxacin; At, azithromycin; Do, doxycyclin. Numbers inside each box indicates the percentage of drug resistance.

Year-wise antibiotic resistance pattern of *V. cholerae* O1 is depicted in Figure 4. *V. cholerae* strains isolated during 1999–2015 have shown different antibiotic resistance patterns at different time periods within the same serogroup [35, 36]. The drug resistance by *V. cholerae* O1 shows: ampicillin (ranges from 53% to 100%), neomycin (50%–90%), nalidixic acid (93%–100%), streptomycin (64%–100%), co-trimoxazole (74%–100%), erythromycin, (63%–100%) and furazolidone (87%–100%). Most of the strains were multidrug resistant and had undergone various changes. Based on the susceptibility patterns *V. cholerae* can be classified into seven groups *viz*. group 1: susceptible to gentamycin throughout the study period; group2: resistance to nalidixic acid throughout the study period; group3: resistant in most of the year except for few years susceptibility (Ampicilin, streptomycin, furazolidone and co-trimoxazole). group 4: equal resistance and susceptible in consecutive 3 year besides regional variation (neomycin). Group 5: susceptible currently to doxycycline, azithromycine and ofloxacine, group 6: geographical variation of drug resistance (chloramphenicol and neomycin) and group 7: chloramphenicol, tetracycline [37], norfloxacin and ciprofloxacin were entirely susceptible except 1 or 2 year resistance (Fig. 4). Though all the strains of O139 were susceptible to nalidixic acid during its emergence in 1995, they became resistant (100%) in 2000 [38]. Variable proportions of ciprofloxacin and norfloxacin (fluoroquinolone) resistant *V. cholerae* O1 and O139 have been reported during 1999–2007 and a combination of norfloxacin and ciprofloxacin resistant *V. cholerae* O1 and O139 existed from 1999 to 2003 [39]. Very interestingly high percentage of polymyxin B (75%) susceptibility was observed in Haitian variant strains isolated during 2014 and 2015 in Puri [33].

In order to prevent potential threats of cholera outbreaks, WHO recommends use of available OCV in conjunction with other preventive and/or control measures in cholera-endemic areas as well as areas at risk of outbreaks [40]. A clinical trial against clinically significant cholera cases in Kolkata has shown 65% protective efficacy over 5 years of an Indian licensed bivalent killed whole cell OCV, Shanchol [41]. The authors have also predicted that this vaccine may exhibit superior result when the vaccine will be administered under real-life condition against undiagnosed cholera. A study conducted in a public health set-up in Odisha suggested that Shanchol is effective (69% with two dose) among rural population and its delivery cost is feasible and affordable for the resource poor countries [42]. It has also been shown that this vaccine is useful and can be widely deployed by government immunisation programmes through public health systems in cholera-endemic areas [24].

## Discussion

This paper is first to provide a comprehensive information on epidemic cholera outbreaks, evolution of genetic variants of *V. cholerae* and trends of antibiogram in Odisha. It has been observed that the state, on average, experiences two cholera outbreaks per year and these are mainly confined to Puri, Khordha and Cuttack districts of the coastal region and Rayagada, Kalahandi and Koraput districts of the Eastern Ghats region. Sporadic cholera cases were reported from all districts while it is endemic in coastal districts [34]. Analysis of data for the last 21 years shows the variation of serotypes and the appearance of new variants of *V. cholerae* O1 at different time periods. The most commonly reported causative agent was *V. cholerae* O1 El Tor biotype and its altered variants, but the frequency of O139 has depleted since 2006.

About seven CT genotypes have been identified due to the mutations in the *ctxB* gene in the world. *V. cholerae* O1 having such mutations are shown to be associated with the epidemic cholera with very high morbidity. In Odisha, since 2002, prevalence of El Tor variant carrying classical *ctxB* gene has increased gradually and replaced the prototype El Tor in 2010. Most of the outbreaks from 2007 to 2012 were caused by the El Tor variant carrying *ctxB1* while the current outbreaks were caused by the Haitian variant in Odisha. Haitian variant was reported for the first time in Odisha from 2007 [14], while it has been prevalent in Kolkata since 2006 [17]. However, the report of *ctxB7* in super-cyclone strains [31] further predicts its origin since 1999 in Odisha. Probably sea water invasion towards inland during super-cyclone might have created an optimum ecological state for the emergence of Haitian variant. Most notably the recent observations in the Odisha isolates of *V. cholerae* O1, harbouring Haitian variant *ctxB* allele, *tcpA* of Haitian allele and high polymyxin B susceptibility are unique genetic and phenotypic change contrast to the El Tor biotype of earlier decades [33].

Cholera has been articulated to maintain its seasonality. Contrast to Bangladesh where two seasonal peaks occur [43], in Odisha cholera outbreaks occur in regular intervals where the peak usually occurs each year corresponding to rainy season. Cholera seasonality begins from pre-monsoon (June) that continues till beginning of winter season (end of October) achieving the peak in September.

In cholera-endemic areas the highest attack rates are in children aged 2–4 years [44]. In contrast, in newly invaded areas by *V. cholerae* new serogroups, the attack rates are similar for all ages. However, the illness is generally first seen in adult men on account of exposure to contaminated food and water [45]. This was evidenced when adults were main victims of cholera caused by the new serogroups of *V. cholerae* O1 El Tor biotype and *V. cholerae* O139 among non-immune population [46]. In Odisha, all the outbreaks were caused by El Tor variants during 2010–2014. During this period El Tor variant replaced completely the predecessor prototype El Tor and new Haitian variant replaced El Tor variant after 2014. The outbreak studies in Odisha reported adults were worst affected by cholera caused by new variants of *V. cholerae* O1 [26, 30, 32, 47] with nearly same proportion of males and females. Furthermore, the post vaccination (OCV) diarrhoea surveillance study conducted in Puri during 2011–2013 revealed 2.5% and 0.9% incidence of *V. cholera* among adults and children respectively (unpublished data). Therefore, like new serogroups, preponderance of adults affected by new variants in cholera-endemic areas speculates pre-existing immunity is poorly expressed. However, proper epidemiological study is needed to confirm this speculation. Besides lack of immunity factor, poor hygiene, lack of safe drinking-water and improper disposal of waste and excreta assist for new infection.

In tribal areas of Odisha, adults were more affected by cholera [26]. Contamination of water is the main cause of transmission of cholera. People usually go to their paddy field for farming and drink water from river, streams and other sources of environmental surface water [26]. Lack of health awareness, illiteracy, unhygienic conditions and deep faith on traditional healers for primary treatment are the other additional factors for origin and spread of cholera with high risk of mortality.

The present report reveals that the ecological condition of coastal and southern tribal districts favours the survival of *V. cholerae* in the aquatic environment during inter-epidemic periods. Phenotypic and genotypic analysis of the clinical and environmental *V. cholerae* strains has led to hypothesise that the aquatic environment is the initial source of infection [48] and therefore use of ground water might be a risk factor, especially during flood and cyclone compared to normal periods.

Although some of the districts have not reported cholera outbreaks during the period under report, its presence cannot be refuted. Documentation and reporting system of diarrhoea morbidity and mortality might not be adequate and prompt enough for several reasons in the state. These include limited disease surveillance, inadequate laboratory capacity, especially at the peripheral healthcare centres and reluctance to acknowledge the problem by authorities for fear of societal repercussion [9]. This might be the cause of under reporting in the affected areas and such situation is true in many other cholera-endemic countries. Rapid diagnostic tests and advanced molecular techniques can be used for effective epidemiological surveillances and track the origin and dissemination of strains causing cholera for adopting appropriate control and prevention measures.

Antibiotics have been used as an adjunct to hydration treatment for cholera since 1964. By decreasing the duration of diarrhoea and stool volume, antibiotics result in more rapid recovery and shorter lengths of inpatient stay, both of which contribute to optimising resource utilisation in an outbreak setting. The observations in the present study with respect to drug resistance illustrate that *V. cholerae* has shown resistance to different recommended drugs including tetracycline, ciprofloxacin and norfloxacin at some point of time and emergence and persistence of multidrug resistance in *V. cholerae* strains limits the therapeutic potential of the drugs. Development of drug resistance might be due to the widespread and irrational use of antibiotics or antibiotics prophylaxis for household contacts of cholera patients during the epidemics [49, 50]. However, further research is urged to understand this hypothesis of drug resistance development. Since emergence and re-emergence of drug resistance in *V. cholerae* unpredictability confuses physicians to select appropriate antibiotics, our study emphasises mandatory monitoring to update epidemiology of antibiotics resistance to prescribe appropriate drug therapies.

Shanchol, a bivalent OCV has been proved to be effective at controlling outbreaks of cholera in Guinea and rural Haiti [51–53]. An efficacy trial of this vaccine in a public health set-up has shown encouraging results in preventing the cholera in Satyabadi block of Odisha [42]. Health officials and policy-makers may plan to implement OCV as an effective component of control and prevention measure of cholera in conjunction with intervention measures as recommended by WHO [54] to reduce the morbidity and mortality in resource poor settings like Odisha.

## Conclusion

In conclusion, Odisha is an endemic home for cholera that is largely caused by *V. cholerae* O1, Ogawa El Tor biotype and its recent variants. Concurrently, emergence of multiple drug resistance is making the infections harder to treat and increasing the risk of spread. Transmission of *V. cholerae* through contaminated water underscores the urgent need for evidence based water, sanitation and hygiene interventions. The efficacy trial of OCV (Shanchol) has revealed convincing results which can be used to immunise the vulnerable population and prevent the cholera outbreaks.

The emergence of new variants and continuous changes of phenotypic characters in *V. cholerae* creates several questions to disclose the mystery of these evolutionary alterations. The emergence of *ctxB7* during super-cyclone 1999 gave us a clue and tempted to hypothesise that changed ecosystem due to hits of cyclones may result for emergence of these new evolutionary variations in *V. cholerae*. Coastal Odisha being the place of sitting duck for extreme cyclones, thus coastal Odisha can be thought as one of the birth place for many evolutionary events of *V. cholerae*. However, a full-fledged integrated study is suggested to understand this hypothesis.

The key epidemiological information of cholera contained in this paper suggests for constant surveillance in other parts of Odisha, India and around the globe for control of cholera outbreaks.

## Data Availability

All relevant data are within the paper and its supporting information files.
